# Supplementary material for: Quantifying Spatial Heterogeneity of Tumor-Infiltrating Lymphocytes to Predict Survival of Individual Cancer Patients
Source: J Pers Med. 2022 Jul 7;12(7):1113. doi: 10.3390/jpm12071113 (PMC9317291; doi:10.3390/jpm12071113)
Supplement: Supplementary file 1 [file jpm-12-01113-s001.zip › jpm-1775220-supplementary-Figures S1-S7.zip!/SuppFigure S1.pdf]

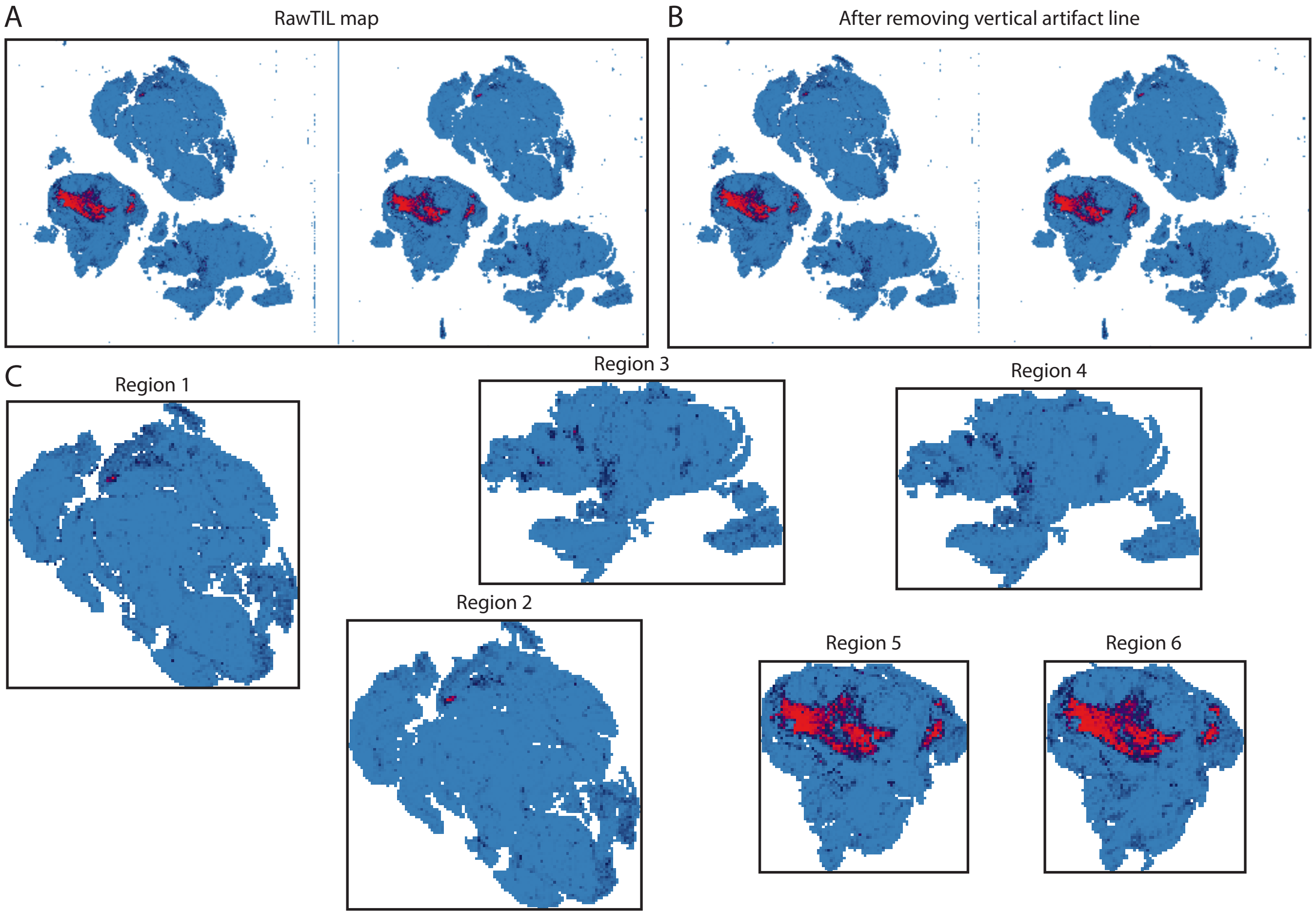
A
RawTIL map
B
After removing vertical artifact line
C
Region 1
Region 3
Region 4
Region 2
Region 5
Region 6
