# Supplementary material for: Quantifying Spatial Heterogeneity of Tumor-Infiltrating Lymphocytes to Predict Survival of Individual Cancer Patients
Source: J Pers Med. 2022 Jul 7;12(7):1113. doi: 10.3390/jpm12071113 (PMC9317291; doi:10.3390/jpm12071113)
Supplement: Supplementary file 1 [file jpm-12-01113-s001.zip › jpm-1775220-supplementary-Figures S1-S7.zip!/SuppFigure S2.pdf]

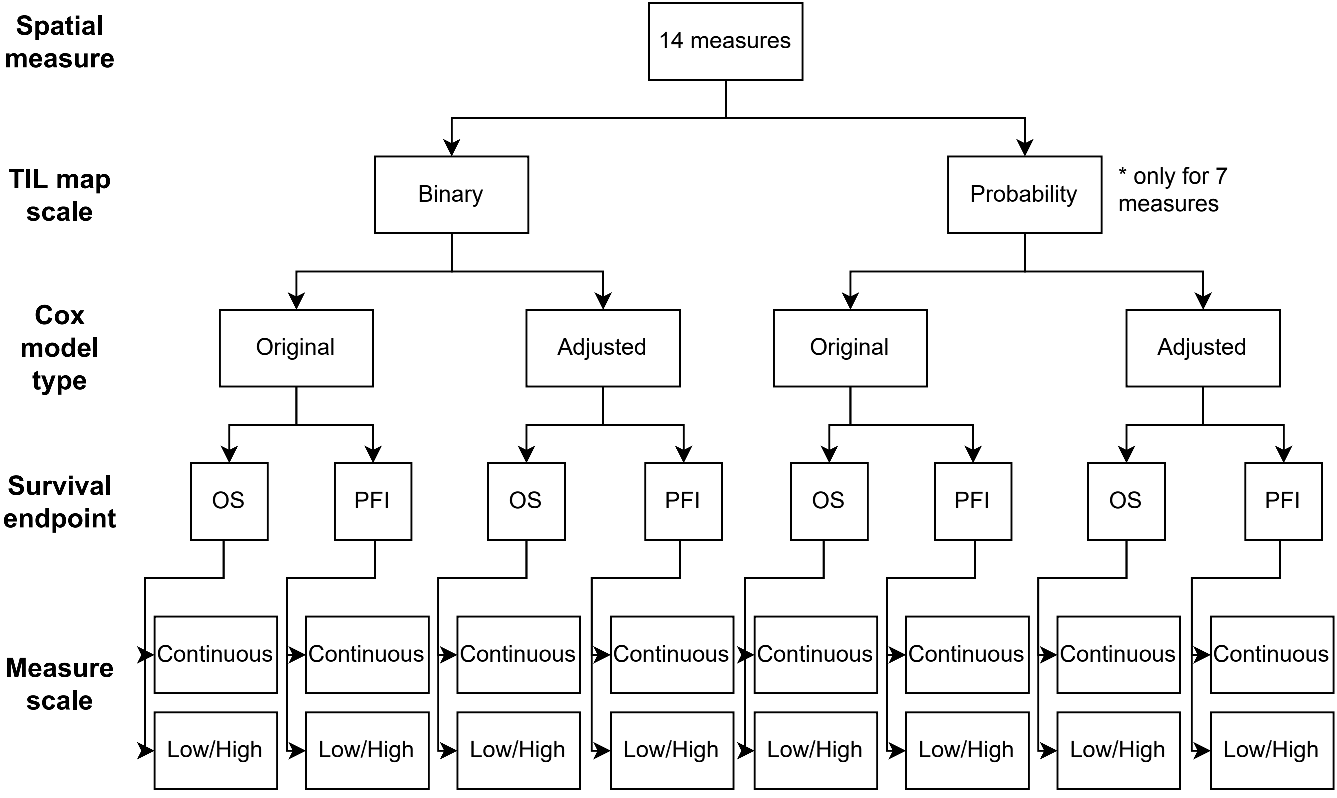
Spatial measure
TIL map scale
Cox model type
Survival endpoint
Measure scale
14 measures
Binary
Probability
* only for 7 measures
Original
Adjusted
Original
Adjusted
OS
PFI
OS
PFI
OS
PFI
OS
PFI
Continuous
Low/High
