# Supplementary material for: Quantifying Spatial Heterogeneity of Tumor-Infiltrating Lymphocytes to Predict Survival of Individual Cancer Patients
Source: J Pers Med. 2022 Jul 7;12(7):1113. doi: 10.3390/jpm12071113 (PMC9317291; doi:10.3390/jpm12071113)
Supplement: Supplementary file 1 [file jpm-12-01113-s001.zip › jpm-1775220-supplementary-Figures S1-S7.zip!/SuppFigure S3.pdf]

A

Binary TIL maps

	GLCM M1	GLCM M2	Spatial Chaos	Ripley F	Ripley G	Ripley L	Spatial autocorrelation	Degree centrality	Average Clustering	Closeness centrality	Ball Hall	Banfeld Raftery	C-index	Det. ratio
acc	0.000	0.000	2.174	4.348	4.348	4.348	4.348	4.348	4.348	4.348	4.348	4.348	6.522	4.348
blca	0.559	0.559	0.559	0.000	0.000	0.000	0.000	0.000	0.000	0.000	0.000	0.000	0.000	0.000
brca	0.188	0.188	0.658	0.940	0.940	0.940	0.940	0.940	0.940	0.940	0.846	0.940	1.692	0.846
cesc	0.000	0.000	0.000	0.377	0.377	0.377	0.377	0.377	0.377	0.377	0.377	0.377	0.755	0.377
coad	0.461	0.461	0.691	0.691	0.691	0.691	0.691	0.691	0.691	0.691	9.677	9.677	9.908	9.677
esca	0.000	0.000	0.000	0.000	0.000	0.000	0.000	0.000	0.000	0.000	0.000	0.000	0.000	0.000
hnsc	0.000	0.000	0.000	0.446	0.446	0.446	0.446	0.446	0.446	0.446	0.446	0.446	0.670	0.446
kirc	0.000	0.000	0.000	0.000	0.000	0.000	0.000	0.000	0.000	0.000	0.000	0.000	0.000	0.000
lihc	0.000	0.000	0.000	0.549	0.549	0.549	0.549	0.549	0.549	0.549	0.549	0.549	0.549	0.549
luad	0.428	0.428	0.428	0.214	0.214	0.214	0.214	0.214	0.214	0.214	0.214	0.214	0.428	0.214
lusc	1.037	1.037	1.037	0.000	0.000	0.000	0.000	0.000	0.000	0.000	0.000	0.000	0.000	0.000
meso	1.149	1.149	1.149	1.149	1.149	1.149	1.149	1.149	1.149	1.149	1.149	1.149	3.448	1.149
ov	0.000	0.000	0.000	0.943	0.943	0.943	0.943	0.943	0.943	0.943	0.943	0.943	2.830	0.943
paad	0.000	0.000	0.000	0.000	0.000	0.000	0.000	0.000	0.000	0.000	0.000	0.000	0.000	0.000
prad	0.000	0.000	0.000	0.000	0.000	0.000	0.000	0.000	0.000	0.000	2.500	2.500	2.500	2.500
read	0.641	0.641	1.282	0.641	0.641	0.641	0.641	0.641	0.641	0.641	0.641	0.641	1.282	0.641
sarc	0.000	0.000	0.000	0.392	0.392	0.392	0.392	0.392	0.392	0.392	0.392	0.392	1.176	0.392
skcm	0.231	0.231	0.462	0.231	0.231	0.231	0.231	0.231	0.231	0.231	0.231	0.231	1.155	0.231
stad	0.000	0.000	0.000	0.270	0.270	0.270	0.270	0.270	0.270	0.270	0.270	0.270	0.539	0.270
tgct	10.738	10.738	10.738	0.000	0.000	0.000	0.000	0.000	0.000	0.000	0.000	0.000	0.000	0.000
thym	0.000	0.000	0.000	0.000	0.000	0.000	0.000	0.000	0.000	0.000	0.000	0.000	0.000	0.000
ucec	0.000	0.000	0.000	0.000	0.000	0.000	0.000	0.000	0.000	0.000	0.000	0.000	0.000	0.000
uvm	0.000	0.000	0.000	0.000	0.000	0.000	0.000	0.000	0.000	0.000	0.000	0.000	0.000	0.000

B

TIL maps with probabilities

	GLCM M1	GLCM M2	Spatial Chaos	Ball Hall	Banfeld Raftery	C-index	Det. ratio
acc	0.000	0.000	0.000	4.348	4.348	6.522	4.348
blca	0.559	0.559	0.559	0.000	0.000	0.000	0.000
brca	0.188	0.188	0.188	0.846	0.940	1.316	0.846
cesc	0.000	0.000	0.000	0.377	0.377	0.755	0.377
coad	0.461	0.461	0.461	9.677	9.677	9.908	9.677
esca	0.000	0.000	0.000	0.000	0.000	0.000	0.000
hnsc	0.000	0.000	0.000	0.446	0.446	0.670	0.446
kirc	0.000	0.000	0.000	0.000	0.000	0.000	0.000
lihc	0.000	0.000	0.000	0.549	0.549	1.099	0.549
luad	0.214	0.214	0.214	0.214	0.214	0.642	0.214
lusc	1.037	1.037	1.037	0.000	0.000	0.000	0.000
meso	100.000	100.000	1.149	1.149	1.149	3.448	100.000
ov	0.000	0.000	0.000	0.943	0.943	3.774	0.943
paad	100.000	100.000	0.000	0.000	0.000	0.000	100.000
prad	0.000	0.000	0.000	2.500	2.500	2.500	2.500
read	0.641	0.641	0.641	0.641	0.641	0.641	0.641
sarc	0.000	0.000	0.000	0.392	0.392	0.784	0.392
skcm	0.231	0.231	0.231	0.231	0.231	0.693	0.231
stad	0.000	0.000	0.000	0.270	0.270	0.539	0.270
tgct	10.738	10.738	10.738	0.000	0.000	0.000	0.000
thym	0.000	0.000	0.000	0.000	0.000	0.000	0.000
ucec	0.000	0.000	0.000	0.000	0.000	0.000	0.000
uvm	0.000	0.000	0.000	0.000	0.000	0.000	0.000
