# Supplementary material for: Quantifying Spatial Heterogeneity of Tumor-Infiltrating Lymphocytes to Predict Survival of Individual Cancer Patients
Source: J Pers Med. 2022 Jul 7;12(7):1113. doi: 10.3390/jpm12071113 (PMC9317291; doi:10.3390/jpm12071113)
Supplement: Supplementary file 1 [file jpm-12-01113-s001.zip › jpm-1775220-supplementary-Figures S1-S7.zip!/SuppFigure S4.pdf]

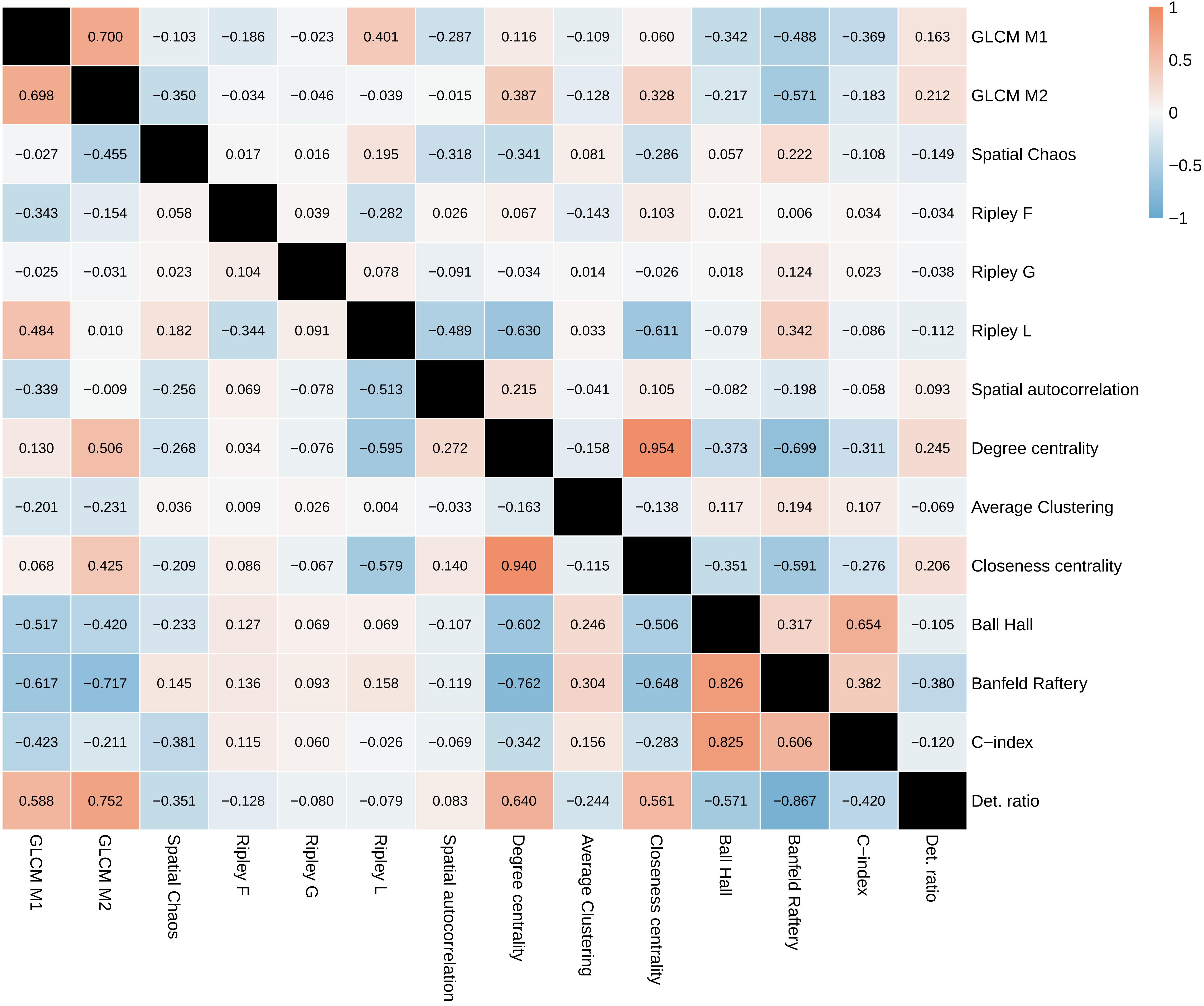
0.700 −0.103 −0.186 −0.023 0.401 −0.287 0.116 −0.109 0.060 −0.342 −0.488 −0.369 0.163 GLCM M1
0.698 −0.350 −0.034 −0.046 −0.039 −0.015 0.387 −0.128 0.328 −0.217 −0.571 −0.183 0.212 GLCM M2
−0.027 −0.455 0.017 0.016 0.195 −0.318 −0.341 0.081 −0.286 0.057 0.222 −0.108 −0.149 Spatial Chaos
−0.343 −0.154 0.058 0.039 −0.282 0.026 0.067 −0.143 0.103 0.021 0.006 0.034 −0.034 Ripley F
−0.025 −0.031 0.023 0.104 0.078 −0.091 −0.034 0.014 −0.026 0.018 0.124 0.023 −0.038 Ripley G
0.484 0.010 0.182 −0.344 0.091 −0.489 −0.630 0.033 −0.611 −0.079 0.342 −0.086 −0.112 Ripley L
−0.339 −0.009 −0.256 0.069 −0.078 −0.513 0.215 −0.041 0.105 −0.082 −0.198 −0.058 0.093 Spatial autocorrelation
0.130 0.506 −0.268 0.034 −0.076 −0.595 0.272 −0.158 0.954 −0.373 −0.699 −0.311 0.245 Degree centrality
−0.201 −0.231 0.036 0.009 0.026 0.004 −0.033 −0.163 −0.138 0.117 0.194 0.107 −0.069 Average Clustering
0.068 0.425 −0.209 0.086 −0.067 −0.579 0.140 0.940 −0.115 −0.351 −0.591 −0.276 0.206 Closeness centrality
−0.517 −0.420 −0.233 0.127 0.069 0.069 −0.107 −0.602 0.246 −0.506 0.317 0.654 −0.105 Ball Hall
−0.617 −0.717 0.145 0.136 0.093 0.158 −0.119 −0.762 0.304 −0.648 0.826 0.382 −0.380 Banfeld Raftery
−0.423 −0.211 −0.381 0.115 0.060 −0.026 −0.069 −0.342 0.156 −0.283 0.825 0.606 −0.120 C−index
0.588 0.752 −0.351 −0.128 −0.080 −0.079 0.083 0.640 −0.244 0.561 −0.571 −0.867 −0.420 Det. ratio
GLCM M1
GLCM M2
Spatial Chaos
Ripley F
Ripley G
Ripley L
Spatial autocorrelation
Degree centrality
Average Clustering
Closeness centrality
Ball Hall
Banfeld Raftery
C−index
Det. ratio
1
0.5
0
−0.5
−1
