# Supplementary material for: Quantifying Spatial Heterogeneity of Tumor-Infiltrating Lymphocytes to Predict Survival of Individual Cancer Patients
Source: J Pers Med. 2022 Jul 7;12(7):1113. doi: 10.3390/jpm12071113 (PMC9317291; doi:10.3390/jpm12071113)
Supplement: Supplementary file 1 [file jpm-12-01113-s001.zip › jpm-1775220-supplementary-Figures S1-S7.zip!/SuppFigure S5.pdf]

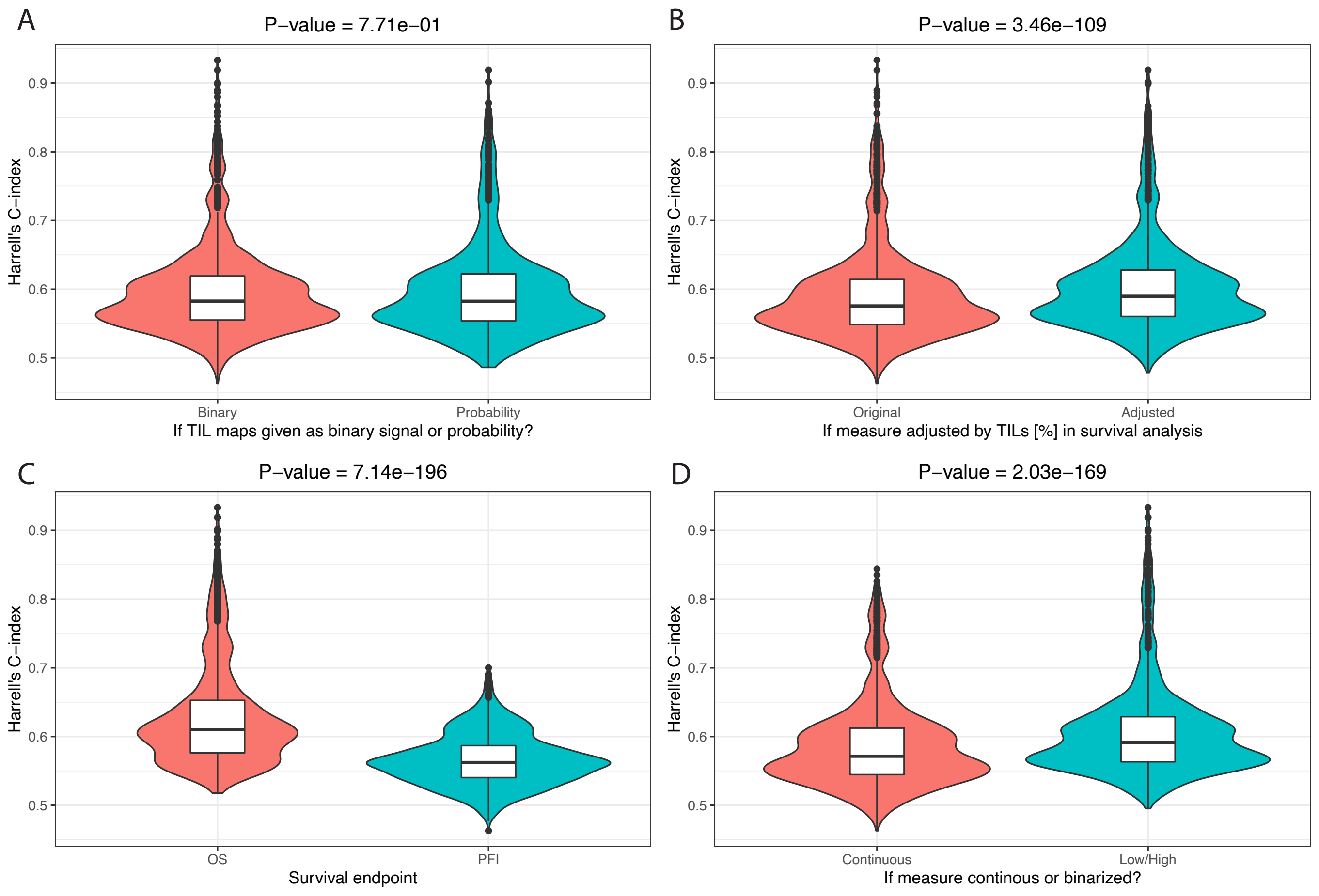

A
P−value = 7.71e−01
Harrell's C−index
0.9
0.8
0.7
0.6
0.5
Binary
Probability
If TIL maps given as binary signal or probability?
B
P−value = 3.46e−109
Harrell's C−index
0.9
0.8
0.7
0.6
0.5
Original
Adjusted
If measure adjusted by TILs [%] in survival analysis
C
P−value = 7.14e−196
Harrell's C−index
0.9
0.8
0.7
0.6
0.5
OS
PFI
Survival endpoint
D
P−value = 2.03e−169
Harrell's C−index
0.9
0.8
0.7
0.6
0.5
Continuous
Low/High
If measure continous or binarized?
