# Supplementary material for: Quantifying Spatial Heterogeneity of Tumor-Infiltrating Lymphocytes to Predict Survival of Individual Cancer Patients
Source: J Pers Med. 2022 Jul 7;12(7):1113. doi: 10.3390/jpm12071113 (PMC9317291; doi:10.3390/jpm12071113)
Supplement: Supplementary file 1 [file jpm-12-01113-s001.zip › jpm-1775220-supplementary-Figures S1-S7.zip!/SuppFigure S6.pdf]

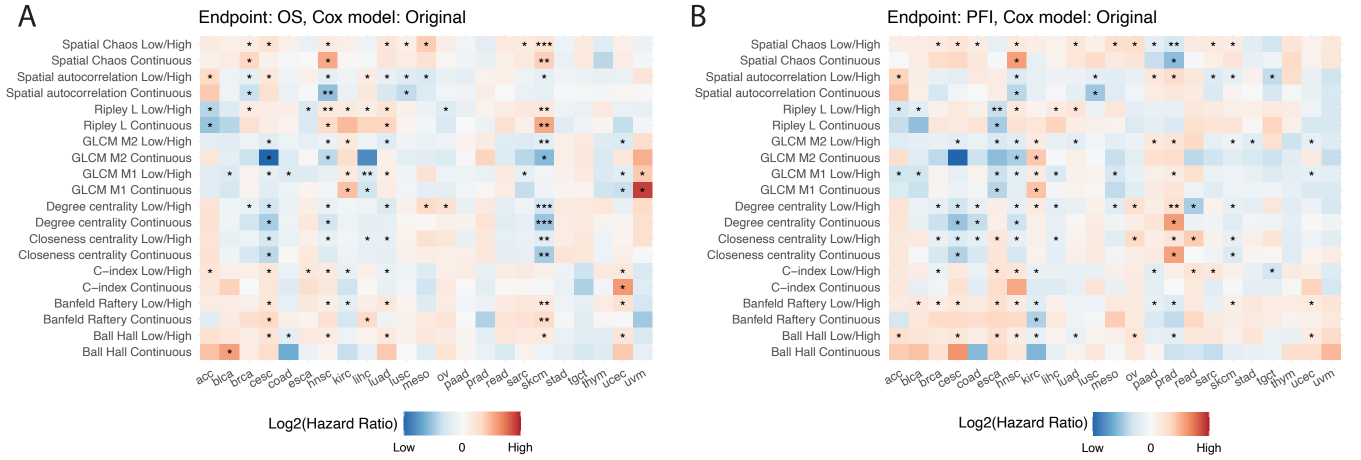
A
Endpoint: OS, Cox model: Original
B
Endpoint: PFI, Cox model: Original
Spatial Chaos Low/High
Spatial Chaos Continuous
Spatial autocorrelation Low/High
Spatial autocorrelation Continuous
Ripley L Low/High
Ripley L Continuous
GLCM M2 Low/High
GLCM M2 Continuous
GLCM M1 Low/High
GLCM M1 Continuous
Degree centrality Low/High
Degree centrality Continuous
Closeness centrality Low/High
Closeness centrality Continuous
C−index Low/High
C−index Continuous
Banfeld Raftery Low/High
Banfeld Raftery Continuous
Ball Hall Low/High
Ball Hall Continuous
acc blca brca cesc coad esca hnsc kirc lihc luad lusc meso ov paad prad read sarc skcm stad tgct thym ucec uvm
Log2(Hazard Ratio)
Low
0
High
