# Supplementary material for: Quantifying Spatial Heterogeneity of Tumor-Infiltrating Lymphocytes to Predict Survival of Individual Cancer Patients
Source: J Pers Med. 2022 Jul 7;12(7):1113. doi: 10.3390/jpm12071113 (PMC9317291; doi:10.3390/jpm12071113)
Supplement: Supplementary file 1 [file jpm-12-01113-s001.zip › jpm-1775220-supplementary-Figures S1-S7.zip!/SuppFigure S7.pdf]

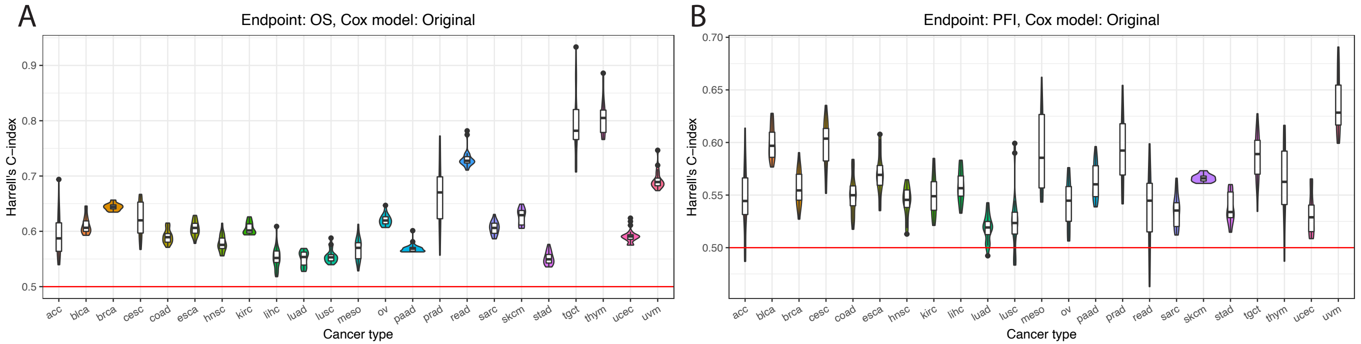
A
Endpoint: OS, Cox model: Original
Harrell's C−index
0.5
0.6
0.7
0.8
0.9
Cancer type
acc blca brca cesc coad esca hnsc kirc lihc luad lusc meso ov paad prad read sarc skcm stad tgct thym ucec uvm
B
Endpoint: PFI, Cox model: Original
Harrell's C−index
0.50
0.55
0.60
0.65
0.70
Cancer type
acc blca brca cesc coad esca hnsc kirc lihc luad lusc meso ov paad prad read sarc skcm stad tgct thym ucec uvm
